# Consumption of Ultra-Processed Foods Is Inversely Associated with Adherence to the Mediterranean Diet: A Cross-Sectional Study

**DOI:** 10.3390/nu14102073

**Published:** 2022-05-15

**Authors:** Monica Dinu, Marta Tristan Asensi, Giuditta Pagliai, Sofia Lotti, Daniela Martini, Barbara Colombini, Francesco Sofi

**Affiliations:** 1 Department of Experimental and Clinical Medicine, University of Florence, 50134 Florence, Italy; mtristanasensi@gmail.com (M.T.A.); giuditta.pagliai@unifi.it (G.P.); sofia.lotti@unifi.it (S.L.); barbara.colombini@unifi.it (B.C.); francesco.sofi@unifi.it (F.S.); 2 Department of Food, Environmental and Nutritional Sciences (DeFENS), University of Milan, 20122 Milan, Italy; daniela.martini@unimi.it; 3 Unit of Clinical Nutrition, Careggi University Hospital, 50134 Florence, Italy

**Keywords:** ultra-processed foods, NOVA classification, NFFQ, Mediterranean diet, Medi-Lite

## Abstract

Information on the consumption of ultra-processed foods (UPF) in relation to an adherence to the Mediterranean diet (MD) is limited. Our aim was to assess UPF consumption in a group of Italian adults and to evaluate the relationship with the MD adherence. A total of 670 participants (median age: 30 years) were included in the analysis. The consumption of UPF was assessed through the NOVA Food Frequency Questionnaire (NFFQ). Adherence to the MD was assessed through the Medi-Lite score. The percentage of UPF in the diet was 16.4% corresponding to 299 g of UPF per day. These amounts were significantly (*p* < 0.05) higher in men than in women and came mainly from ready-to-eat meals or pre-packaged bread, bread alternatives, pizza, frozen potato chips (24.5% of total UPF intake), pre-packaged biscuits and sweets (20.7%), soft drinks (15.8%), and dairy products such as flavored yogurt (12%). As to the MD adherence, a significant inverse association between the Medi-Lite score and the percentage of UPF in the diet (R = −0.35; *p* < 0.001) was observed. Participants with a low adherence to the MD had a significantly higher contribution of UPF in the diet (22.2%) compared to those with a moderate (16.2%) and high (12.6%) adherence. In terms of individual UPF, the largest difference between low and high MD adherents was observed for pre-packaged biscuits and sweets, soft and energy drinks, sausages and other reconstituted meat products, and pre-packaged bread and bread alternatives. These results suggest that public health strategies are needed to implement more effective actions to promote healthy eating habits in the population.

## 1. Introduction

Ultra-processed foods (UPF) are defined within the NOVA classification system, which groups foods according to the extent and purpose of industrial processing [1]. They are “formulations of ingredients, mostly for industrial use only, derived from a series of industrial processes” [2]. Examples of UPF are pre-packaged frozen meals, ready-to-eat meals, fast food, mass-produced bread, savory snacks, breakfast cereals with added ingredients, reconstituted meat products, instant soups and noodles, and soft and sweetened beverages. 

Available literature suggests that higher UPF consumption is associated with a lower diet quality, leading to diets high in calories, free sugars, fat, and salt, and low in dietary fiber [3]. This, in turn, may be associated with a worse cardio—metabolic risk profile and an increased risk of cardiovascular disease, cerebrovascular disease, depression, and all-cause mortality, as suggested by a recent meta-analysis by our group [4]. It is also known that increased UPF intake can replace unprocessed foods and freshly prepared meals and dishes [2], which are the basis of traditional dietary patterns recognized to promote long and healthy lives [5,6,7]. 

The Mediterranean diet (MD) is one of the most studied dietary patterns in the scientific community. Despite its health benefits, widely documented in epidemiological and clinical studies [8,9], many Mediterranean countries are experiencing a progressive shift away from this dietary pattern [10]. In this regard, some recent studies have suggested an association between MD adherence and UPF consumption, but evidence is still limited [11,12,13,14,15]. Furthermore, available studies have not used specifically validated tools to estimate UPF consumption and this could potentially lead to a misinterpretation of the associations found [16]. The aim of this study was to evaluate UPF consumption in a group of Italian adults and to assess the possible relationship between MD adherence and UPF consumption using validated tools specifically designed for these purposes.

## 2. Materials and Methods

### 2.1. Study Design and Data Collection

A web survey was conducted between January and October 2021. Data were collected anonymously from an online questionnaire prepared on SurveyMonkey (www.surveymonkey.com, accessed on 11 May 2022) [17], which is a free tool with an easy-to-use web interface. To enroll as many participants as possible, the questionnaire was disseminated and shared with a link among personal and non-personal contacts using advertisements in local media, social media, and on websites. Before starting the questionnaire, participants were asked to read the project information sheet, which contained an explanation of the study’s objectives. Then, participants were asked to complete a brief questionnaire focused on sociodemographic characteristics (age, sex, marital status, education level, weight, and height), and two validated questionnaires suitable for collecting information on UPF consumption and MD adherence. Marital status was categorized as unmarried/single and married/partner, and education level was categorized as primary/secondary school, high school, and university. Body mass index (BMI) was calculated as weight in kilograms divided by the square of height in meters for each participant. Participants were classified as overweight if their BMI ranged between 25–30 kg/m^2^, and obese if their BMI was 30 kg/m^2^ or more. This study was conducted according to the guidelines laid down in the Declaration of Helsinki and all procedures involving human subjects were approved by the Ethics Committee of the University of Florence, Florence, Italy [n 199/2022, protocol 11917]. Informed consent was obtained from all participants. 

### 2.2. NOVA Food Frequency Questionnaire (NFFQ) 

The NFFQ is a validated questionnaire specifically designed to estimate food intake according to NOVA groups in the Italian adult population [18]. The questionnaire includes 94 items divided into nine categories: (1) fruits and nuts; (2) vegetables and legumes; (3) cereals and tubers; (4) meat and fish; (5) milk, dairy products, and eggs; (6) oils, fats, and seasonings; (7) sweets and sweeteners; (8) beverages; and (9) other. For each item, the participant should indicate the frequency of consumption and usual portion size considering the diet in a typical month over the past 12 months. Answers correspond to one out of ten options: (1) “never or less than once a month”; (2) “one to three times a month”’; (3) “once a week”; (4) “twice a week”; (5) “three times a week”; (6) “four times a week”; (7) “five times a week”; (8) “six times a week”; (9) “every day”; and (10) “if every day, how many times a day?”. Because all 94 items included in the FFQ were pre-categorized according to the NOVA classification, each food fell into one of the following categories: unprocessed and minimally processed food or beverage (MPF); processed culinary ingredients (PCI); processed food or beverage (PF); and UPF. 

When processing the NFFQ data, the amount of food consumed is calculated in grams per week and in grams per day for each food item and category. Then, the proportion of MPF, PCI, PF, and UPF in the diet is determined by calculating the weight ratio. As previously reported, the weight ratio is considered rather than the energy ratio because it allows for a better accounting for processed foods that do not provide energy (e.g., artificially sweetened beverages) and non-nutritional factors related to food processing (e.g., newly formed contaminants, food additives and alterations in the structure of raw foods) [18]. Finally, because PCI products are not intended to be consumed alone as food, but are supposed to be used to prepare and season other foods, they are grouped with PF. 

### 2.3. The Medi-Lite Adherence Score 

Adherence to MD was assessed through the validated Medi-Lite questionnaire, developed by Sofi et al. [19]. The questionnaire includes nine domains, based on daily and/or weekly consumption of fruits, vegetables, cereals, legumes, fish, meat and meat products, dairy products, alcohol, and olive oil. For fruits, vegetables, cereals, legumes, and fish (typical foods of MD), two points are given for the highest consumption category, one point for the medium category and zero points for the lowest category. For olive oil, two points are given for regular use, one for frequent use, and zero for occasional use. On the other hand, for meat and meat products and dairy products (foods not typical of MD), two points are given for the lowest category of consumption, one point for the medium category, and zero for the highest category. Finally, for alcohol consumption, two points are given for the medium category, one for the lowest category, and zero for the highest category. The questionnaire score ranges from 0 to 18, where the highest value corresponds to the highest adherence to the MD.

### 2.4. Statistical Analysis 

Descriptive statistics were used to analyze and report the data. Results were reported as mean ± standard deviation (SD), median and range, or geometric mean with 95% confidence intervals (CIs), as appropriate. Categorical variables were presented in terms of frequencies and percentages. The Mann—Whitney test was used for comparisons between women and men, while the Chi—Square test was used to test for proportions. The correlation between the percentage of UPF in the diet and the Medi-Lite score was estimated using the Spearman (R) test. 

The possible relationship between UPF consumption and MD adherence was analyzed by grouping the participants according to UPF contribution in the diet (1st tertile ≤ 10%; 2nd tertile = 10–19%; 3rd tertile ≥ 19%), and by MD adherence into low (1st tertile = Medi-Lite score < 9), moderate (2nd tertile = Medi-Lite score between 9 and 11), and high (3rd tertile = Medi-Lite score > 11) adherence. Then, a general linear model adjusted for age, sex, BMI, education level, marital status, and daily food intake was conducted to compare dietary habits according to the percentage of UPF in the diet, and daily UPF intake according to the MD adherence. Since these tests assume normal data distribution, non-distributed data were transformed into logs and further analyses were performed with the processed data. However, to facilitate interpretation, the log data were again converted to the original scale (antilog) and presented as geometric means with 95% CIs.

Finally, to evaluate the influence of individual UPF on Medi-Lite score, a linear regression model was performed, and results were expressed as regression coefficient (b) ± SE. The b coefficient estimated in the linear regression analysis indicates the expected mean change in Medi-Lite score associated with 1-unit change in the independent variables. *p*-values  < 0.05 were considered statistically significant. The statistical package IBM SPSS Statistics for Macintosh, version 28.0 (IBM Corp., Armonk, NY, USA) was used.

## 3. Results

A total of 670 participants (70.4% women) with a mean age of 35.8 years were included in the analysis. Table 1 reports their socio-demographic characteristics, according to sex. Overall, the study population was highly educated and more than half of the participants were single. Compared to men, women had significantly (*p* < 0.001) lower BMI and lower prevalence of overweight and obesity (21.1% vs. 40.9%). No significant differences were observed for education level or marital status. 

### 3.1. Nova Food Frequency Questionnaire (NFFQ)

The percentage of UPF in the diet was 16.4 ± 10.4%, with significantly (*p* < 0.05) lower values in women (15.6 ± 10%) than in men (18.2 ± 11.1%). No significant differences were observed according to BMI, education level, and marital status. In terms of daily UPF intake, the percentage of UPF in the diet corresponded to 299.0 ± 233.5 g/day in the total sample, 285.5 ± 231.0 g/day in women, and 331.2 ± 236.7 g/day in men. The food categories that contributed most to this amount were ready-to-heat meals or pre-packaged bread, bread alternatives, pizza, and frozen potato chips (24.5% of total UPF intake), pre-packaged sweets and biscuits (20.7%), soft drinks (15.8%), flavored yogurt (12.0%), followed by ready-to-eat vegetables and legumes (10.6%), dairy products and meat substitutes (8%), sausages and other reconstituted meat and fish products (5.8%), and fats and seasonings (2.5%). The contribution of individual UPF, according to sex, is reported in Supplementary Table S1. Significant differences between sex were observed for ready-to-heat pasta/gnocchi, sausages and other reconstituted meat and fish products, and for alcoholic beverages (e.g., liquor), with higher percentages in men than in women. 

Food consumption (g/day) according to the percentage of UPF in the diet is reported in Table 2. After adjustment for possible confounding factors such as age, sex, and daily food intake, participants in the highest UPF tertile showed a lower consumption of fruits, nuts, and vegetables than those in the first tertile, and a higher consumption of cereals and tubers, fats and seasonings, sweets and sweeteners, beverages, and dairy substitutes. For meat and fish, the total consumption did not differ significantly between groups, but subjects in the highest UPF tertile consumed more meat and poultry and less fish and seafood. A similar trend was observed for beverages, with subjects in the highest UPF tertile reporting a higher consumption of soft and energy drinks, and subjects in the lowest UPF tertile reporting a higher consumption of tea and coffee. No significant differences were observed for milk and dairy products, except for cheese.

### 3.2. Medi-Lite and UPF Consumption

**Figure 1:**
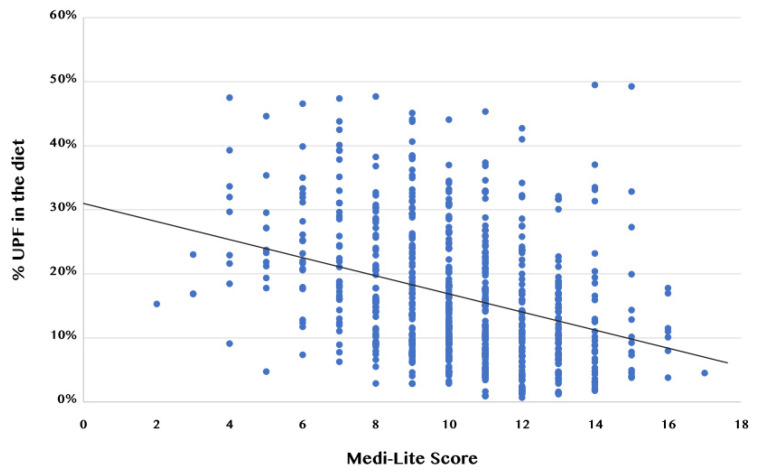
Correlation between Medi-Lite score and the percentage of UPF in the diet. Legend: UPF: ultra-processed foods.

Regarding MD adherence, the mean Medi-Lite score was 10.3 ± 2.5, with no significant differences (*p* = 0.151) between women (10.4 ± 2.4) and men (10 ± 2.6). Correlation analyses showed a significant inverse association between the Medi-Lite score and percentage of UPF in the diet (R = −0.35; *p* < 0.001) (Figure 1), which was confirmed when women (R = −0.35; *p* < 0.001) and men (R = −0.36; *p* < 0.001) were analyzed separately.

**Figure 2:**
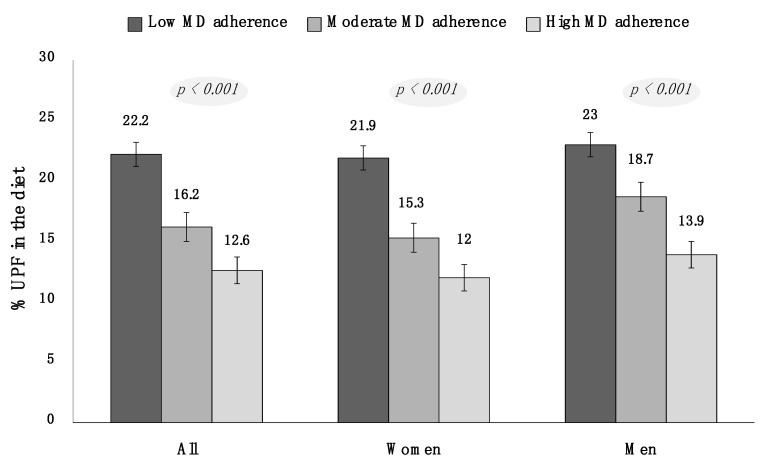
Percentage of UPF in the diet according to MD adherence. Legend: MD: Mediterranean diet; UPF: ultra-processed foods.

To better explore the possible relationship between MD adherence and UPF consumption, three adherence groups to MD (low, moderate, and high) were considered, and the mean percentage of total UPF in these groups was calculated. As showed in Figure 2, subjects with low MD adherence had a significantly higher contribution of UPF in the diet (22.2 ± 10.3%) than those with moderate (16.2 ± 9.8%) and high (12.6 ± 9.5%) adherence. This result was confirmed when women and men were analyzed separately. 

Regarding individual UPF, their intake in terms of daily amounts was significantly higher in subjects with a low MD adherence for most of the items considered, with the largest differences observed for pre-packaged biscuits and other sweets, soft and energy drinks, sausages and other reconstituted meat products, and pre-packaged bread and bread alternatives (Table 3). 

Finally, to evaluate the possible influence of individual UPF on Medi-Lite score, a linear regression analysis with the Medi-Lite score as a dependent variable was performed. As shown in Table 4, sausages and other reconstituted meat products, pre-packaged pizza, potatoes, biscuits and sweets, and soft and energy drinks were revealed to influence the Medi-Lite score significantly and negatively after an adjustment for age, sex, BMI, education level, marital status, and the total food consumed.

## 4. Discussion

In this study we used a validated questionnaire to assess UPF consumption in relation to an adherence to the MD in a group of adult subjects living in the Mediterranean area. In our study group, we observed that subjects reporting a low adherence to MD were also those showing a higher consumption of UPF in their diet. In fact, an increased intake of UPF was associated with a significantly lower intake of some typical products of the MD such as fruits, vegetables, nuts and fish, and a higher intake of meat, fats, seasonings, and sugary products. Furthermore, we observed that some UPF, such as sausages and other reconstituted meat products, pre-packaged pizza, frozen potato chips, industrial biscuits and sweets, and soft and energy drinks, were shown to influence the adherence score to MD.

Since Monteiro and colleagues proposed the NOVA classification to identify the level of food processing [20], several studies have investigated UPF consumption and its effects on health. A meta-analysis by our group recently reported a significantly increased risk of the occurrence of major chronic diseases in association with a higher UPF intake [4]. In terms of daily UPF consumption, a significant increase has been reported worldwide in recent decades. Data from most of the industrialized countries show a range of percentages from 20–50% of UPF present in the diet of the general population. To date, countries bordering the Mediterranean Sea have shown a lower UPF consumption in contrast to Western countries, but the intake of UPF is increasing rapidly, leading to a gradual displacement of long-established diets. In our sample population, the percentage of UPF in the diet was 16% of the total energy intake, which corresponded to a daily consumption of almost 300 g of UPF. These values are slightly higher than those reported also in Italy but in the Southern part by Bonaccio et al., (10% of total energy intake, corresponding to 182 g/day) [13] but substantially lower compared with other countries such as Spain (24%) [21], France (36%) [22], the United Kingdom (57%) [23], or the United States (58%) [24]. Such a difference among countries, in particular between Mediterranean and non-Mediterranean countries, let us to hypothesize that the traditional dietary patterns of the Mediterranean basin could have influenced the findings. 

Currently, a higher adherence to MD is known to reduce the risk of all-cause mortality, cardiovascular diseases, coronary heart disease, myocardial infarction, overall cancer incidence, neurodegenerative diseases, and diabetes [8]. In line with the literature [11,12,13,14,15], the results obtained from this study confirm a significant inverse association between UPF consumption and adherence to MD, highlighting that higher UPF consumption is associated with a lower adherence to MD. This could be explained as a nutritional transition from fresh meals and dishes that are part of the traditional cuisine towards a higher intake of ready-to consume and hyper-palatable food and beverages products. Indeed, an impact on the intake of some of the foods known to be the basis of MD was reported due to the high UPF consumption. Specifically, participants with greater UPF intake reported a lower intake of fruits, vegetables, nuts, and fish, and a higher intake of cereals, fats, meat, seasonings, and sweets in their diets, in contrast to participants with a lower UPF consumption. Similar findings were found in other studies of Mediterranean populations, such as Spain [11] or France [22]. Furthermore, UPF consumption has not only impacted eating habits, but also beverage quality, with a higher consumption of soft and energy beverages and a lower consumption of water, tea, and coffee in participants with a greater UPF consumption [14,15,22]. 

To better understand the association between UPF intake and MD adherence in our study population, we also investigated the possible influence of individual UPF on the MD adherence score, observing that the consumption of sugary products, processed meats, soft and energy drinks, and pre-packaged potatoes and pizza negatively influenced the MD adherence score. Interestingly, most of these foods were major contributors to UPF consumption in our population and are present in all countries of the world, supporting the hypothesis of a nutritional transition from traditional diets such as MD to Westernized dietary patterns due to an increased UPF consumption. There is an urgent need to raise awareness on the negative health effects of excessive UPF consumption and new public health strategies to prevent the progressive loss of traditional diets. 

The present study has several limitations that deserve discussion. First, the cross-sectional design does not allow us to establish any cause-effect relationship. Prospective cohort studies are needed to confirm these preliminary findings. Second, the use of self-administered online questionnaires may have led to recall bias and misclassification. The emergency response due to the COVID-19 pandemic did not allow us to conduct another type of study with a stronger statistical power and less biases. On the other hand, several strengths are present since a validated questionnaire specifically designed to estimate food intake according to NOVA classification was used, avoiding the misclassification of foods into UPF categories. Furthermore, the variables used in the main analyses included the proportion, by weight, of UPF in the diet. This approach is more appropriate than the use of energy proportion, as it takes a better account of non-nutritional factors pertaining to food processing (e.g., neo-formed contaminants, additives, alterations to the structure of raw foods). 

## 5. Conclusions

In conclusion, in our study population of middle-aged Italian adults we were able to observe an inverse relation between adherence to MD and UPF consumption. In considering the over-consumption of UPF as an important risk factor for non-communicable diseases, overweightness and obesity, our results reinforce the importance of public health strategies to improve the population’s health by promoting MD and limiting the intake of UPF, which is also proposed by the World Health Organization (WHO). Examples of such strategies could be taxation, the regulation of marketing, or the control of food labeling standards, some of which have already shown good results [25,26]. Moreover, particular attention should be paid to the consumption of specific UPF (sugary products, processed meats, soft and energy beverages, pre-packaged potatoes, and pizza) due to their association with a low adherence to MD. 

## Figures and Tables

**Table 1:** Characteristics of the study population.

	All (*n* = 670)	Women (*n* = 472)	Men (*n* = 198)	*p*-Value
Age, year	35.8 ± 13.4	35.3 ± 13	36.9 ± 14.1	0.160
Body weight, kg	66.5 ± 14.1	61.5 ± 11.7	78.6 ± 11.9	<0.001
BMI, kg/m^2^	23.2 ± 4	22.6 ± 4.1	24.7 ± 3.3	<0.001
BMI ≥ 25 kg/m^2^	176 (26.3)	95 (20.1)	81 (40.9)	<0.001
Education level				
Secondary school	23 (3.4)	15 (3.2)	8 (4)	0.744
High school	289 (43.1)	198 (41.9)	91 (46)	0.384
University	358 (53.4)	259 (54.9)	99 (50)	0.285
Marital status				
Single	372 (55.5)	253 (53.6)	119 (60.1)	0.144
Married/partner	256 (38.2)	189 (40)	67 (33.8)	0.155
Divorced/widowed	42 (6.3)	30 (6.4)	12 (6.1)	0.886

Legend: BMI = Body Mass Index. Data are reported as mean ± standard deviation or number and percentage (%), as appropriate

**Table 2:** Food consumption (g/day) according to the percentage of UPF in the diet.

	%UPF in the Diet			
	<10% *n* = 222	10–19% *n* = 227	>19% *n* = 221	*p* Trend ^†^
**Fruits and nuts**	275.9 (251.1–302.8)	173.1 (157.9–190)	117.1 (106.6–128.8) *	<0.001
Fruits	258.3 (233.9–285.4)	159.0 (144.2–175.4)	104.5 (94.5–115.5) *	<0.001
Dried and syrup fruits	3.3 (2.6–4.2)	3.4 (2.7–4.3)	4.2 (3.3–5.3)	0.304
Nuts	9.8 (8.3–11.4)	7.6 (6.5–8.9)	6.5 (5.5–7.7) *	0.002
**Vegetables and legumes**	387.2 (353.9–423.3)	298.9 (273.7–326.4)	231.6 (211.7–253.4) *	<0.001
Vegetables	360.0 (326.7–396.6)	276.4 (251.4–304.0)	211.0 (191.3–232.8) *	<0.001
Legumes	20.4 (17.9–23.3)	20.0 (17.4–23.1)	22.8 (19.6–26.6)	0.432
**Cereals and tubers**	218.8 (206.2–232.1)	268.0 (252.9–284)	299.2 (281.7–317.3) *	<0.001
Grains (e.g., rice, spelt, barley, wheat)	19.0 (16.9–21.4)	18.3(16.3–20.6)	14.9 (13.1–16.8) *	0.010
Pasta, bread, and pizza	146.5 (136.2–157.4)	183.1 (170.4–196.6)	220.3 (204.8–237.0) *	<0.001
Potatoes and tubers	37.6 (33.9–41.8)	49.3 (44.5–54.4)	50.1 (45.2–55.6) *	<0.001
Breakfast cereals	9.9 (8.2–12.0)	8.0 (6.7–9.5)	9.0 (7.5–10.7)	0.246
**Meat and fish**	105.6 (97.9–114)	117.8 (109.3–127)	119.2 (110.4–128.8)	0.054
Meat and poultry	54.6 (49.8–59.8)	69.5 (63.5–76.0)	78.9 (72.1–86.5) *	<0.001
Fish and seafood	45.0 (40.8–49.7)	40.7 (36.9–44.8)	33.6 (30.4–37.2) *	<0.001
**Milk, dairy products, and eggs**	130.7 (115.5–148.0)	138.2 (122.4–156.3)	122.2 (107.8–138.7)	0.389
Milk and milk beverages (e.g., probiotic milk)	74.6 (62.1–89.7)	62.5 (52.2–74.7)	60.7 (50.4–73.0)	0.244
Yogurt	38.2 (32.7–44.7)	42.4 (36.7–49.1)	41.3 (35.3–48.3)	0.620
Cheese	26.3 (23.0–30.0)	32.3 (28.3–36.8)	32.5(28.5–37.1) *	0.040
Eggs	14.7 (13.6–16.0)	14.1 (13.1–15.3)	12.9 (11.8–14.0) *	0.067
**Oil, fats, and seasonings**	38.7 (36.6–40.9)	43.8 (41.4–46.2)	51.5 (48.7–54.4) *	<0.001
Olive oil and vegetable oils	25.7 (24.7–26.8)	25.8 (24.8–26.8)	25.2 (24.2–26.2)	0.692
Other fats (e.g., butter, margarines)	1.3 (1.1–1.5)	1.7 (1.5–1.9)	1.9 (1.7–2.2) *	<0.001
Sauces	11.3 (10.0–12.7)	14.1 (12.5–15.8)	21.4 (19.0–24.1) *	<0.001
**Sweets and sweeteners**	39.7 (35.6–44.2)	66.6 (60.0–74.2)	73.6 (66.0–82.1) *	<0.001
Biscuits, cakes, snacks, and ice-cream	29.4 (26.3–32.9)	50.5 (45.2–56.3)	59.8 (53.4–67) *	<0.001
Chocolate, spreads, and candies	6.9 (5.9–8.1)	9.4 (8.1–11.0)	9.4 (8.0–11.0) *	0.006
Sugar	2.8 (2.1–3.7)	2.4 (1.8–3.1)	3.0 (2.3–3.9)	0.499
**Beverages**	246.7 (225.0–270.2)	273.7 (250.1–299.5)	308.0 (280.6–337.6) *	0.004
Tea and coffee	139.9 (124.6–157.3)	120.7 (107.4–135.4)	97.7 (86.7–110.1) *	<0.001
Fruit and vegetable juice	42.1 (32.7–54.1)	57.1 (47.0–69.3)	61.1 (51.0–73.3) *	0.057
Soft and energy drinks	27.1 (22.0–33.4)	41.5 (35.7–48.2)	87.1 (76.3–99.4) *	<0.001
Alcoholic beverages	54.7 (45.6–65.6)	67.3 (55.8–81.0)	49.1 (40.8–59.1)	0.060
**Other**	19.1 (14.3–25.6)	31.4 (24.5–40.4)	45.0 (35.2–57.5) *	<0.001
Plant-based dairy substitutes	22.0 (15.4–31.5)	42.3 (31.1–57.5)	49.4 (37.3–65.4) *	0.003
Plant-based meat substitutes	11.6 (9.2–14.6)	11.9 (10.0–14.2)	10.5 (8.9–12.5)	0.596

Legend: UPF = ultra-processed foods. Data are reported as geometric mean and 95% confidence interval (CI). ^†^ Adjusted for age, sex, BMI, education level, marital status, and total food consumed (g/day). * *p* < 0.05 for differences between the 1st and the 3rd tertile adjusted for age, sex, BMI, education level, marital status, and total dietary intake (g food/day)

**Table 3:** UPF intake (g/day) according to the MD adherence.

	Adherence to the MD			
	Low *n* = 143	Moderate *n* = 325	High *n* = 203	*p* Trend ^†^
**Vegetables and legumes UPF**	38.2 (30.8–47.6)	43.8 (37.8–50.7)	34.8 (28.5–42.4)	0.167
Ready-to-heat vegetables and legumes (with added ingredients)	38.2 (30.8–47.6)	43.8 (37.8–50.7)	34.8 (28.5–42.4)	0.167
**Cereals and tubers UPF**	70.5 (59.1–84.3)	43.2 (38.7–48.7)	33.5 (28.8–39) *	<0.001
Ready-to-heat pasta/gnocchi dishes	17.9 (14.9–21.6)	17.5 (15.3–19.9)	15.1 (12.5–18.1)	0.363
Pre-packaged breads, buns, and bread alternatives	18.2 (15.1–22.0)	13.8 (12.2–15.6)	11.9 (10.1–13.9) *	0.004
Pre-packaged pizza, focaccia, sandwich, and savory pies	33.5 (27.4–41.0)	24.5 (21.2–28.3)	25.6 (20.6–31.8)	0.043
Pre-packaged instant rice, soups, noodles	13.4 (10.5–17.1)	12.3 (10.3–14.7)	11.8 (9.2–15.0)	0.754
Breakfast cereals and energy bars (with added sugar)	7.7 (5.9–9.9)	6.5 (5.5–7.6)	6.8 (5.4–8.5)	0.547
Pre-packaged potatoes (e.g., frozen potato chips)	17.9 (15.8–20.3)	14.9 (13.6–16.4)	13.9 (12.2–15.9) *	0.022
**Meat and fish UPF**	21.5 (18.8–24.5)	15.7 (14.3–17.3)	14.9 (13.0–17) *	<0.001
Nuggets, sticks, sausages, burgers, and other reconstituted meat products	18.9 (16.6–21.5)	13.9 (12.6–15.2)	13.0 (11.4–14.9) *	<0.001
Fish nuggets, fish sticks, and other reconstituted fish products	7.9 (6.9–9)	8.4 (7.6–9.3)	7.9 (7.0–9.1)	0.626
**Milk and dairy products UPF**	20.7 (16.3–26.3)	23.4 (19.9–27.5)	18.9 (14.9–26.9)	0.302
Milk beverages (e.g., probiotic milk with added sugar)	15.8 (11.5–21.9)	22.6 (17.7–28.9)	18.3 (13.4–25.1)	0.205
Fruit or flavored yogurts (e.g., vanilla flavored)	25.0 (20.0–31.1)	27.1 (23.5–31.2)	24.3 (19.8–29.8)	0.632
Melted cheese (also used to stuff sandwich)	3.4 (2.9–4.0)	2.9 (2.5–3.3)	3.2 (2.6–3.8)	0.240
**Fats and seasonings UPF**	6.7 (5.5–8.1)	5.6 (4.9–6.5)	5.5 (4.6–6.6)	0.309
Margarines and other spreads	0.8 (0.4–1.6)	0.9 (0.5–1.7)	0.9 (0.4–1.6)	0.940
Pre-packaged or instant sauces (e.g., mayonnaise, ketchup, meat sauce)	7.0 (5.8–8.6)	5.7 (4.9–6.5)	5.5 (4.6–6.5)	0.138
**Sweets and sweeteners UPF**	54.6 (46.8–63.7)	35.8 (32.5–39.6)	29.8 (63.7–33.8) *	<0.001
Pre-packaged biscuits, cakes, snacks, and ice-cream	39.4 (33.1–46.9)	25.0 (22.3–28)	21.9 (18.9–25.5) *	<0.001
Chocolate, spreads (e.g., nut spread), and candies	10.2 (8.4–12.4)	8.8 (7.7–10.0)	7.2 (6.1–8.5) *	0.033
**Beverages UPF**	57.9 (44.6–75.3)	35.3 (29.5–42.1)	25.0 (19.8–31.5) *	<0.001
Soft and energy drinks (e.g., iced tea, coke)	72.2 (59.0–89.0)	51.4 (44.4–59.4)	45.2 (36.9–55.4) *	0.005
Alcoholic beverages (e.g., rum, gin, spirits)	5.7 (4.3–7.4)	4.9 (4.1–5.7)	4.8 (4.0–5.8)	0.589
**Other UPF**	29.3 (19.1–44.8)	27.4 (22.2–33.9)	33.7 (25.3–44.8)	0.527
Plant-based dairy substitutes (e.g., soy yogurt, tofu)	35.2 (21.3–58.4)	33.7 (26.4–42.9)	49.3 (35.3–68.9)	0.195
Plant-based meat substitutes (e.g., veggie burger)	11.8 (8.2–16.9)	10.9 (9.2–12.8)	10.1 (8.1–12.8)	0.783

Legend: MD: Mediterranean diet; UPF: ultra-processed foods. Data are reported as geometric mean and 95% confidence interval (CI). ^†^ Adjusted for age, sex, BMI, education level, marital status, and total food consumed (g/day). * *p* < 0.05 for differences between low and high adherence to the MD adjusted for age, sex, BMI, education level, marital status, and total dietary intake (g food/day).

**Table 4:** Linear regression analysis relating UPF intake (g/day) and Medi-Lite score.

	Medi-Lite Score			
	Model 1		Model 2 ^a^	
	ß (SE)	*p*-Value	ß (SE)	*p*-Value
Pre-packaged breads, buns, and bread alternatives	−0.035 (0.003)	0.369	-	-
Pre-packaged pizza, focaccia, sandwich, and savory pies	−0.116 (0.003)	0.003	−0.156 (0.003)	<0.001
Pre-packaged potatoes (e.g., frozen potato chips)	−0.143 (0.005)	<0.001	−0.201 (0.005)	<0.001
Nuggets, sticks, sausages, burgers, and other reconstituted meat products	−0.204 (0.004)	<0.001	−0.243 (0.004)	<0.001
Pre-packaged biscuits, cakes, snacks, and ice-cream	−0.089 (0.002)	0.021	−0.149 (0.002)	<0.001
Chocolate, spreads (e.g., nut spread), and candies	−0.017 (0.006)	0.669	-	-
Soft and energy drinks (e.g., iced tea, coke)	−0.098 (0.001)	0.011	−0.150 (0.001)	<0.001

^a^ Model 2 includes age, sex, BMI, education level, marital status, and total food consumed (g/day) as covariates.

## Data Availability

Additional data are available from the corresponding author on reasonable request.

## Supplementary Materials

The following supporting information can be downloaded at: https://www.mdpi.com/article/10.3390/nu14102073/s1, Table S1: Contribution (%) of individual foods to the total intake of UPF.
