# Supplementary material for: Consumption of Ultra-Processed Foods Is Inversely Associated with Adherence to the Mediterranean Diet: A Cross-Sectional Study
Source: Nutrients. 2022 May 15;14(10):2073. doi: 10.3390/nu14102073 (PMC9147239; doi:10.3390/nu14102073)
Supplement: Supplementary file 1 [file nutrients-14-02073-s001.zip › nutrients-1716933-supplementary.pdf]

Supplementary Table S1. Contribution (%) of individual foods to the total intake of UPF

	All (n=670)	Women (n=472)	Men (n=198)	p-value
**Vegetables and legumes UPF**	10.6 ± 16.2	9.8 ± 10	12.4 ± 18	0.104
Ready-to-heat vegetables and legumes (with added ingredients)	10.6 ± 16.2	9.8 ± 10	12.4 ± 18	0.104
**Cereals and tubers UPF**	24.5 ± 17.7	24.7 ± 17.7	23.9 ± 18	0.513
Ready-to-heat pasta/gnocchi dishes	3.1 ± 5.9	3.4 ± 6.3	2.4 ± 5	0.005
Pre-packaged breads, buns, and bread alternatives	9.3 ± 11.9	9.3 ± 11.3	9.5 ± 13.2	0.315
Pre-packaged pizza, focaccia, sandwich, and savory pies	5.2 ± 8.7	5.1 ± 8.4	5.6 ± 9.2	0.331
Pre-packaged instant rice, soups, noodles	1.1 ± 3.2	1.1 ± 2.9	1.2 ± 3.7	0.846
Breakfast cereals and energy bars (with added sugar)	2.4 ± 5.5	2.5 ± 5.2	2.1 ± 6.1	0.067
Pre-packaged potatoes (e.g., frozen potato chips)	3.3 ± 5.1	3.3 ± 5.2	3.2 ± 4.7	0.993
**Meat and fish UPF**	5.8 ± 7	5.3 ± 6.7	6.8 ± 7.4	0.005
Nuggets, sticks, sausages, burgers, and other reconstituted meat products	5.0 ± 6.5	4.6 ± 6.4	5.7 ± 6.6	0.009
Fish nuggets, fish sticks, and other reconstituted fish products	0.8 ± 2	0.7 ± 1.7	1.1 ± 2.6	0.045
**Milk and dairy products UPF**	12.0 ± 17	12.6 ± 18	10.6 ± 14.2	0.326
Milk beverages (e.g., probiotic milk with added sugar)	3.4 ± 10.6	3.9 ± 12	2.1 ± 5.9	0.362
Fruit or flavored yogurts (e.g., vanilla flavored)	7.9 ± 13.4	7.9 ± 13.6	7.8 ± 13	0.750
Melted cheese (also used to stuff sandwich)	0.7 ± 1.6	0.7 ± 1.6	0.7 ± 1.6	0.459
**Fats and seasonings UPF**	2.5 ± 3.6	2.3 ± 3.3	2.9 ± 4.2	0.077
Margarines and other spreads	0.1 ± 0.3	0.1 ± 0.3	0.1 ± 0.3	0.330
Pre-packaged or instant sauces (e.g., mayonnaise, ketchup, meat sauce)	2.4 ± 3.6	2.3 ± 3.2	2.9 ± 4.2	0.063
**Sweets and Sweeteners UPF**	20.7 ± 16	21.0 ± 16.5	20.1 ± 14.8	0.985
Pre-packaged biscuits, cakes, snacks, and ice-cream	14.7 ± 13.5	14.8 ± 14.1	14.6 ± 12	0.547
Chocolate, spreads (e.g., nut spread), and candies	6.0 ± 8.2	6.2 ± 8.3	5.6 ± 8.0	0.309
**Beverages UPF**	15.8 ± 18.3	15.5 ± 18.6	16.5 ± 17.7	0.125
Soft and energy drinks (e.g., iced tea, coke)	14.2 ± 18.1	14.1 ± 18.3	14.7 ± 17.6	0.343
Alcoholic beverages (e.g., rum, gin, spirits)	1.6 ± 4.2	1.4 ± 4	1.9 ± 4.4	0.001
**Other UPF**	8.0 ± 10.7	8.6 ± 17.6	6.6 ± 15.6	0.051
Plant-based dairy substitutes (e.g., soy yogurt, tofu)	6.5 ± 16	7.0 ± 16.5	5.5 ± 14.7	0.124
Plant-based meat substitutes (e.g., veggie burger)	1.5 ± 4.4	1.6 ± 4.6	1.2 ± 3.7	0.119

Data are reported as mean ± standard deviation
